# Integrating Nurse Practitioners Into Radiation Oncology: One Institution’s Experience

**Published:** 2014-01-01

**Authors:** Genevieve Hollis, Erin McMenamin

**Affiliations:** From Hospital of the University of Pennsylvania, Philadelphia, Pennsylvania

The demand for cancer-related services is soon expected to exceed the number of available oncologists. Factors contributing to this gap include the aging population and the associated increased incidence of cancer diagnosis, expanding treatment options, a growing number of survivors, changes in reimbursement, and fewer physicians specializing in oncology (Erikson, Salsberg, Gaetano, Bruinooge, & Goldstein, 2007; Institute of Medicine [IOM], 2009; Towle et al., 2011). In radiation oncology in particular, the demand for radiation therapy (RT) is expected to increase 10 times faster from 2010 to 2020 than the supply of radiation oncologists (Smith et al., 2010).

Across all settings, nurse practitioners (NPs) have consistently demonstrated their ability to provide safe, efficient, high-quality, well-received cancer care, and they have been acknowledged as critical in addressing this ever-expanding gap (Cunningham, 2004; Erikson et al., 2007; IOM, 2009; Hinkle et al., 2010; Towle et al., 2011). Barriers to autonomous NP practice persist and include a lack of role clarity, practices limiting independent decision-making, a disproportionate involvement in indirect clinical activities, and physician resistance (Chumbler, Geller, & Weier, 2000; Vogel, 2010; Towle et al., 2011; McCorkle et al., 2012; Moote et al., 2012). There is a need for the thoughtful development of creative collaborative models to address these barriers and facilitate NPs’ ability to practice to the fullest extent of their scope, education, and competence, thereby maximizing their ability to deliver excellent, efficient cancer care (Buswell, Ponte, & Shulman, 2009; IOM, 2009; Moote et al., 2012).

Despite the significant number of patients with cancer who receive RT and the many complex symptoms that these patients experience, until relatively recently, radiation oncology had largely been bereft of NPs. Although some literature has described various clinical activities that can be performed by NPs in radiation oncology, there has been little to guide the effective development and integration of these roles into practice (Carper & Haas, 2006; Moote et al., 2012).

In 2008, the department of radiation oncology at the Hospital of the University of Pennsylvania relocated to the much larger Perelman Center for Advanced Medicine (PCAM). The additional technology available in this new setting—including proton therapy in the Roberts Proton Therapy Center—significantly increased the RT options available to a larger number of patients, treatment planning complexity, and clinical research efforts. To meet the challenges associated with this growth, the department engaged in a thoughtful process that ultimately resulted in multiple successful NP practice models. This article summarizes the key elements of the process and outlines future directions.

## Shared Expectations of the NP Role

A critical initial step in the process of developing a practice model that included NPs was the development of a clear understanding, as well as shared expectations, regarding the NP role. Physicians and administrators were briefed on NP education, scope of practice, regulation, reimbursement, and potential outcomes. Shared expectations evolved and included having NPs practice to the fullest extent of their scope of practice, with the majority of their clinical activities being performed independently. This would predictably result in an enhanced capability to accommodate increased patient volumes (Towle et al., 2011; Moote et al., 2012). Another expectation was the ability to capitalize on NPs’ unique knowledge and skills, thereby increasing the breadth, depth, and quality of health promotion and supportive care provided to patients and families. The final expectation was the NPs’ advancement of the department’s academic mission through the pursuit of related professional activities.

To ensure sufficient patient volumes capable of supporting full-time NP positions, facilitate productivity, and enable development and dissemination of clinical expertise, each NP position was aligned with a specific team of physicians (usually disease-based) rather than with individual physicians. Recognizing that one NP model would not meet the unique needs of all patients and teams, the lead NP collaborated with physicians on each team to analyze patient needs across the illness continuum, current gaps in care, and emerging trends (Carper & Haas, 2006). They determined which clinical activities were only within the physicians’ scope of practice (e.g., prescription, planning, and oversight of RT) and which were central to quality resident education.

Through this analysis, clinical activities within the NP’s independent scope of practice that would enhance patient capacity, expand the supportive care services available to patients, and provide opportunities for substantive professional activities were identified. This ultimately resulted in tailored job descriptions for each NP role. Potential productivity and quality care metrics, as well as financial feasibility, were explored. The proposed job descriptions were submitted to departmental leadership for potential approval. Verbal scripts and a brochure on the benefit of physician/NP collaboration were developed to facilitate communication with patients and colleagues, and NP biographies were placed on the departmental website.

## Orientation and Retention

Most oncology NPs are educated in primary care or acute care programs that lack substantive cancer-related didactic and clinical components. They usually acquire cancer-specific knowledge and skills on the job through physician mentoring and self-study. This model results in a significant increase in time until the NPs are competent independent providers (Vogel, 2010; Nevidjon et al., 2010). This approach is inefficient, may inadvertently emphasize a medical model approach to care, and can undermine the NPs’ future credibility with colleagues and patients.

Consequently, the lead NP developed an orientation based upon published oncology NP competencies as well as the knowledge and skill needs of new oncology NPs and radiation oncology nurses (Oncology Nursing Society [ONS], 2007; Rosenzweig et al., 2012; Brunner & Hollis, 2012). The orientation was tailored to each NP’s educational preparation, oncology work experience, and aligned team. Nurse practitioner and physician preceptors established didactic and clinical experiences that facilitated knowledge acquisition and application to a progressively independent clinical practice. New NPs attended selected on-site lectures and completed specific online courses available through the ONS. Although the majority of their clinical time was spent with NP and physician preceptors, new NPs also spent time with other radiation oncology staff (e.g., registered nurses, therapists, dosimetrists, social workers, and dietitians) and colleagues from other departments who frequently refer to radiation oncology (e.g., medical and surgical oncology providers and nurse navigators). The latter were critical for the NPs to establish collegial relationships and learn to navigate the health system.

Given the significant fiscal and human resource investment in NP recruitment and orientation, retention strategies have focused on promoting professional growth and a healthy, respectful work environment. An individualized professional development plan addresses each NP’s ongoing learning needs, formative evaluation and modification of role, and expectations for professional activity. Nurse practitioners are expected to obtain national certification as an Advanced Oncology Certified NP (AOCNP®) through the Oncology Nursing Certification Corporation (ONCC) within 2 years of completing orientation. As appropriate, NPs are encouraged to obtain a post-master’s certificate in oncology nursing and complete ONS’s Radiation Oncology Certificate Program. The lead NP is expected to complete semiannual reviews with input from collaborative physicians, other NPs, registered nurses, and the director of nursing. Monthly NP meetings and faculty meetings with physician colleagues provide opportunities to inform clinical operations. Work schedules are dependent on the needs of the service/department, and clear efforts are made to encourage a healthy work/life balance.

## Metrics

Assessment of clinical activity generally is based on reimbursement data and resource-value units (RVUs). When patients are receiving RT, reimbursement includes professional fees for weekly treatment management. Due to Medicare regulations, NPs currently are not able to independently perform or bill for these professional services. Additionally, no radiation oncology providers can bill for additional professional evaluation and management services during a course of RT or for follow-ups within 90 days of RT completion. Of note, these are the time periods when patients are likely to be symptomatic and apt to benefit from NP interventions. Consequently, reimbursement and RVU data are inadequate in tracking NP activity in radiation oncology.

In collaboration with information technology experts, an alternative method was developed. Data that track clinical activities performed by NPs in "billable" and "nonbillable" time periods are extracted from the electronic medical record, as well as those completed independently or as part of a shared encounter with a physician. The revenue generated from billable independent NP encounters is tracked. Quality-care measures are tracked, including patient satisfaction and medication reconciliation, pain assessment and management, and smoking assessment and cessation counseling. Lastly, professional activities such as presentations, publications, research involvement, quality-improvement projects, and committee participation are also tracked. This information is reported monthly and used to examine trends, formatively evaluate roles, and stimulate future role development.

## Current Status

Six robust and increasingly independent NP models have emerged that facilitate accommodation of increased patient volumes and expand services available to diverse patient populations. These models encompass clinical activities ranging from consultation to supportive care on and after treatment, disease surveillance, survivorship care, and coordination of care.

Nurse practitioners aligned with teams treating high volumes of prostate cancer patients primarily provide lifelong disease surveillance, management of late effects, and survivorship care in independent follow-up clinics. They also provide expert symptom management to patients with other genitourinary cancers, such as bladder and metastatic prostate cancers, during and immediately after treatment.

The priority for the NPs aligned with teams treating patients with high acuity—such as head and neck, lung, and gastrointestinal cancers—is to provide time-intensive, expert supportive care to these patients during weekly on-treatment physician visits. They independently manage the supportive care and ad hoc/urgent patient needs that occur during treatment and in NP follow-up clinics in the weeks immediately after treatment, when toxicities are expected to peak. This comprehensive approach to supportive care is particularly critical, as these patients consistently have a high symptom burden that can negatively impact quality-of-life and treatment outcomes, the latter due to treatment interruptions.

The head and neck cancer service NP independently performs procedures such as nasopharyngolaryngoscopies and placement of Dobhoff feeding tubes and is exploring an independent survivorship clinic. Given the high symptom burden of lung cancer patients presenting to radiation oncology, the NPs aligned with the lung services screen incoming consults. Patients with identified risk factors for complex symptoms are seen by the NP during the consultation for more comprehensive symptom assessment and initiation of early supportive care interventions. Patients with early-stage lung cancer and gastrointestinal cancer are increasingly being seen in independent NP follow-up clinics for disease surveillance and survivorship care.

Timely completion of inpatient consults, particularly for urgent treatment of oncologic emergencies, is extremely challenging for radiation oncology teams with busy outpatient practices. Additionally, patients hospitalized while receiving RT require coordination of care with inpatient teams to maximize symptom management, minimize treatment interruptions, and avoid gaps during transitions in care. Based upon the success of the previous NP models, a new NP position focusing on the inpatient population was developed. This NP triages all requests for inpatient consultation, performs the actual consult with direct physician input as medically indicated, and coordinates care for patients admitted while receiving RT.

The department provides RT services for pediatric patients from Children’s Hospital of Pennsylvania. A significant aspect of the pediatric NP’s role is to coordinate care between two separate health systems. This NP provides critical on-site pediatric clinical expertise in a predominantly adult care setting, strengthening the delivery of pediatric supportive care and ad hoc/urgent care provided within the department.

Collectively, patient, colleague, and administrator satisfaction with care provided by these NPs has been high. As individual NPs have gained clinical experience and confidence, the independent aspects of their clinical practice have progressively increased. The NPs have incorporated a variety of evidence-based health promotion and supportive care interventions into the care provided to patients and families. Nurse practitioner professional activities have included becoming department committee members, leading quality improvement initiatives, participating in the planning and delivery of the radiation oncology course for nurses, receiving academic appointments at local schools of nursing, authoring peer-reviewed publications, presenting at local and national conferences, and initiating NP-led clinical research efforts.

## Future Directions

The process of developing and sustaining creative collaborative models for NPs in radiation oncology continues. The established NP models are continually evaluated, and emerging trends are analyzed to identify further opportunities for independent NP practice. Departmental leadership is pursuing initiatives to address persistent barriers to optimal utilization of NPs, including providing adequate clinical space and decreasing their involvement in indirect clinical activities that can be performed by other staff members. Efforts are under way to expand the tracking of quality-care metrics that capture the NPs’ impact on patient, departmental, and institutional outcomes.

Nurse practitioner retention strategies that support professional development opportunities and a healthy work environment need to be expanded to include formal mentor relationships, protected time for professional activities, and a professional advancement ladder that is tied to compensation. Collaborating with academic institutions and professional organizations in the development of novel education and training programs to ensure ongoing availability of highly qualified NPs in radiation oncology is a long-term imperative of the department as well as the oncology profession as a whole.

## Conclusion

With strong leadership support, utilization of a content expert, and thoughtful planning, this busy academic radiation oncology department has been successful in establishing a structure for the development and integration of NPs. By empowering NPs to practice to the full scope of their education, licensure, and competency, they have been able to positively impact patient care, increase capacity, expand services available to patients, and become professionally productive. Although challenges exist, so do opportunities to fully implement sophisticated NP roles that positively impact patient, departmental, institutional, and professional outcomes. This process could potentially serve as a model for other practices looking to incorporate NPs into their provider groups.

## Acknowledgments

The authors would like to acknowledge Amy Avellino, MS, RN; Steven Hahn, MD; James Metz, MD; and Robert Lustig, MD, for their collaboration and leadership.
